# Altered nasal airflow: an unusual complication following implant surgery in the anterior maxilla

**DOI:** 10.1186/s40729-016-0045-3

**Published:** 2016-03-29

**Authors:** Jan Wolff, K. Hakki Karagozoglu, Jochen H. Bretschneider, Tymour Forouzanfar, Engelbert A. J. M. Schulten

**Affiliations:** 1 Department of Oral and Maxillofacial Surgery/Oral Pathology, VU University Medical Center/Academic Centre for Dentistry Amsterdam (ACTA), Amsterdam, The Netherlands; 2 Department of Otorhinolaryngology-Head and Neck Surgery, VU University Medical Center, P.O. Box 7057, 1007 MB Amsterdam, The Netherlands

## Abstract

Dental implants have been in routine clinical use for over three decades and are a predictable treatment modality. However, as with all other aspects of dentistry, complications occur. A 50-year-old female patient with complaints of a long ongoing unpleasant altered nasal airflow presented herself at the VU University Medical Center Amsterdam. Visual inspection of the right nasal cavity revealed that the apical part of a dental implant placed in the upper right first incisor region had perforated the nasal floor and was partially protruding into the nasal cavity. Subsequent treatment consisted of a transnasal resection of the apical part of the dental implant to the level of the nasal floor. After a 12-month follow-up period, the patient reported having no altered nasal airflow. In conclusion, dental implants protruding into the nasal cavity can cause an alteration to the airflow. Furthermore, a partial removal of the apical part of the dental implant is a viable method of treating dental implants that extend into the nasal cavity.

## Background

Endosseous dental implants are commonly used to rehabilitate fully or partially edentulous patients [1]. The insertion of such implants can in some cases cause complications, especially in the edentulous atrophic maxilla [2–4]. In this paper, an unusual complication of altered nasal airflow after the placement of an endosseous dental implant in the maxilla is presented. Subsequent treatment of the obstructive nasal airflow is described.

## Case presentation

A 50-year-old female patient was referred to the Department of Oral and Maxillofacial Surgery of the VU University Medical Center in Amsterdam with complaints of a long ongoing unpleasant altered nasal airflow after the placement of eight dental implants in the maxilla. Four months prior to implant surgery, a bony augmentation of the atrophic edentulous alveolar crest and a bilateral maxillary sinus floor elevation using autogenous bone harvested from the anterior iliac crest had been performed. Shortly after implant placement, one of the implants placed in the area of the left first incisor had to be removed due to an oronasal fistula and subsequent lack of osseointegration. No further complications such as rhino-sinusitis, nasal discharge, pain, recurrent epistaxis, or headaches were reported.

**Fig. 1:**
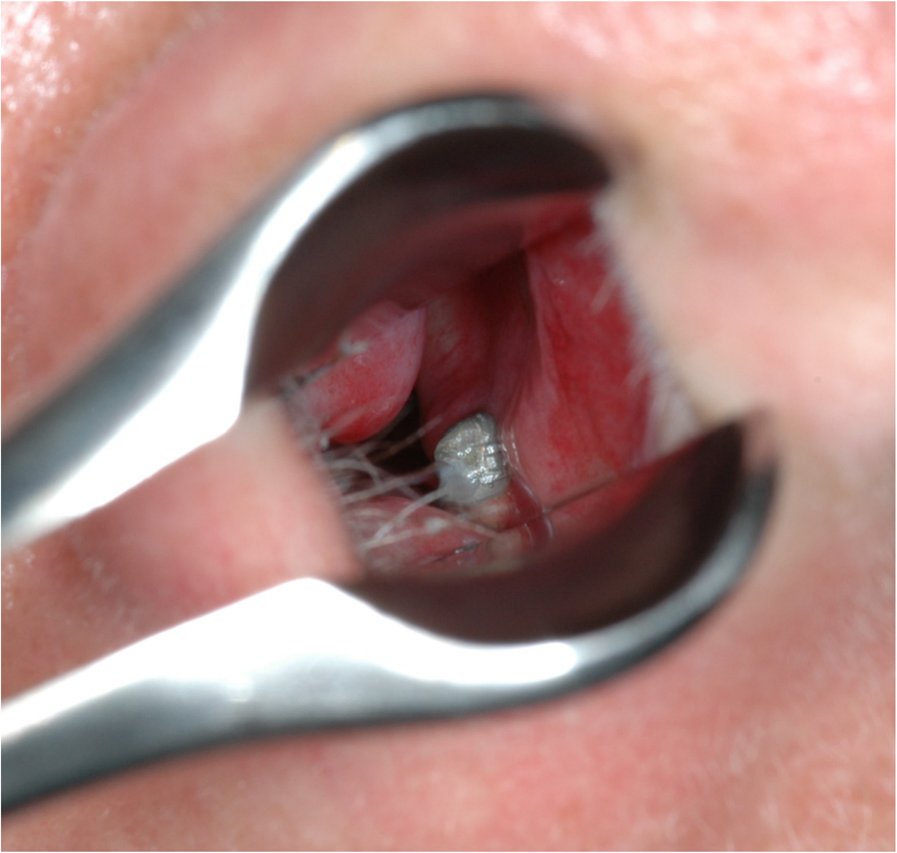
On anterior rhinoscopy, the apical part of the titanium dental implant in the right anterior maxilla was seen in the nasal floor close to the nasal septum

**Fig. 2:**
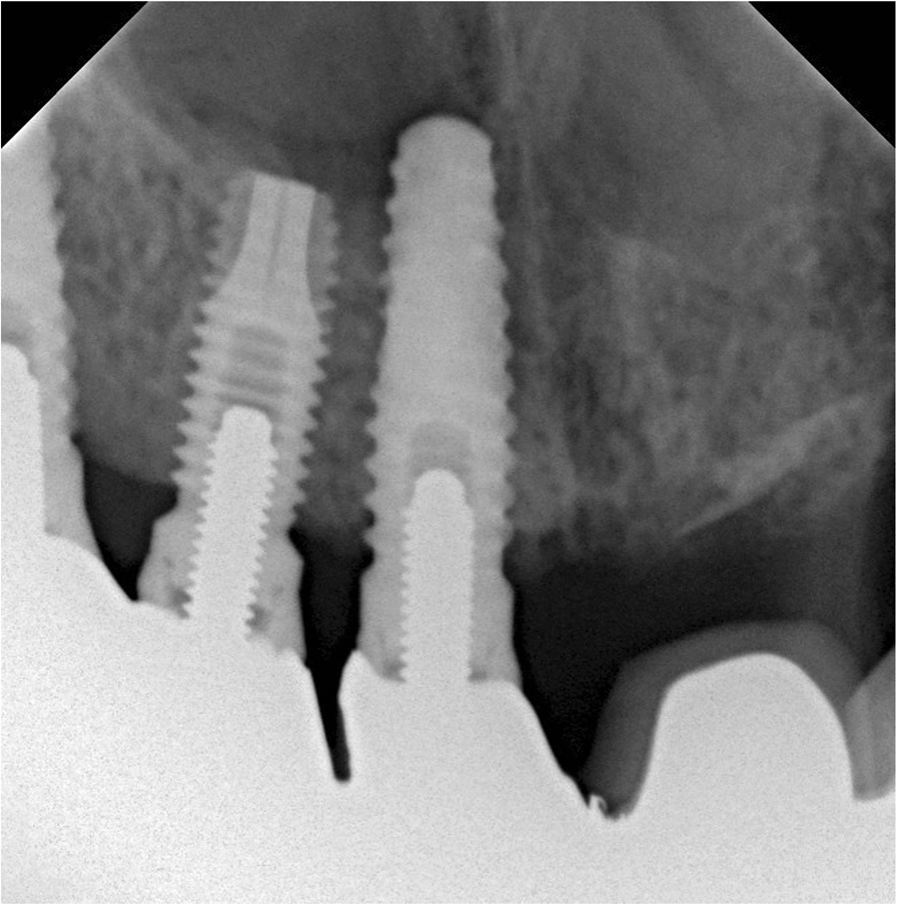
On radiological examination, it was confirmed that the dental implant had perforated the cortical bone of the right nasal floor

However, anterior rhinoscopic examination revealed that the apical part of the dental implant placed in the upper right first incisor region had perforated the nasal floor close to the nasal septum and partially extended into the right nasal cavity (Fig. 1). The mucosa of the left nasal cavity was intact and demonstrated no signs of inflammation. Radiological examination (dental and panoramic radiographs and computer tomography) confirmed that the implant placed in the right first incisor region had perforated the cortical bone of the nasal floor (Fig. 2).

After a discussion with the patient regarding the risks and benefits of surgery, transnasal resection of the apical part of the titanium dental implant in general anesthesia was opted for. During surgery, the floor of the nasal cavity was locally anesthetized and the nasal mucosa surrounding the dental implant was incised and meticulously elevated exposing the nasal floor and the apical part of the perforating dental implant. Under direct vision and adequate sterile saline cooling, the perforating implant was resected to the level of the nasal bone initially using a hard steel fissure burr. The titanium surface was further smoothened with a round diamond burr. All metal debris were carefully removed from the operating site, and the nasal mucosal flap was realigned and sutured using 4-0 Vicryl to provide a watertight mucosal seal. The patient was postoperatively instructed to avoid sneezing and nose blowing and received a broad-spectrum antibiotic (amoxicillin-clavulan acid 500/125 mg three times daily for 5 days).

**Fig. 3:**
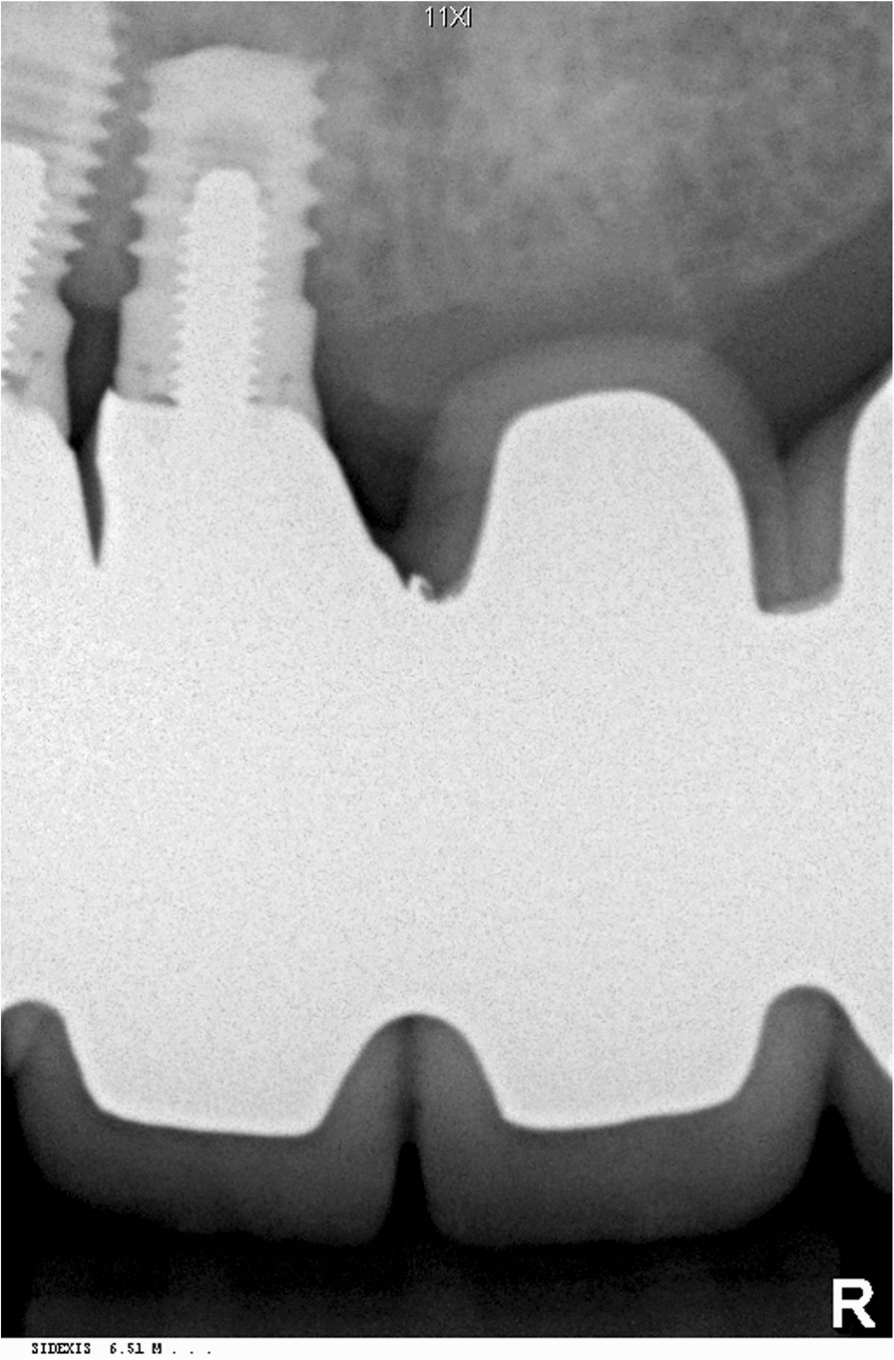
Postoperative radiograph of the resected dental implant in the right anterior maxilla

No complications were apparent during the surgical procedure. Postoperative clinical and radiological examinations demonstrated an intact nasal mucosa and an adequate resection of the dental implant to the level of the nasal floor (Fig. 3). The patient had an uneventful recovery and at 2-, 6-, and 12-month follow-up, she reported having no altered nasal airflow.

### Discussion

Insertion of endosseous dental implants is usually associated with a low incidence of complications and excellent prognosis [1, 2]. However, physiologic changes following tooth loss may complicate or even impede insertion of dental implants in the upper jaw. Furthermore dental implants can only be inserted if there is sufficient bone for adequate stabilization [2–4]. Therefore, in severely atrophied bone conditions, augmentation procedures using autogenous bone grafts or bone substitutes are often required [1–4].

Various short- and long-term complications such as maxillary sinusitis, oroantral fistula, and extrusion of graft material have been reported after implant placement [2–4]. Particularly, dental implants that partially extend into the maxillary sinus or nasal cavity are known to cause complications [2–4]. Furthermore, patients with a predisposition to develop sinusitis are prone for complications after dental implant placement in the maxillary sinus area [1]. Interestingly dental implants that partially extend into the nasal cavity are often asymptomatic and may reside in the nose for many years. However, when complications do occur, unilateral mucopurulent and fetid nasal discharge are the most prevalent symptoms, which can be accompanied by pain, discomfort, headache, or congestion of the affected side. Therefore, patients complaining of nasal discharge after dental implant placement should be thoroughly checked for foreign bodies in their nasal cavities. Differential diagnosis of a unilateral nasal obstruction may also include nasal tumors, nasal polyps, septal deviations, hematomas, and various infections [5].

Minimal invasive treatment strategies for dental implants residing in the nasal cavity as described in this study have to the best of our knowledge not often been described. A more invasive removal of the complete dental implant in the presented case would have had a negative effect on the load bearing during mastication because of its strategic position in the maxilla supporting the fixed bridge construction. Furthermore, an explantation through the oral cavity could have created an oronasal communication and compromised mucosal blood supply resulting in mucosal recession with a negative outcome on esthetics and peri-implant supporting tissue. Therefore, a partial removal of the apical part of the dental implant using a transnasal approach was opted for.

## Conclusions

In conclusion, dental implants protruding into the nasal cavity can cause alterations to the airflow. Dental implants partially residing in the nasal cavity can be minimal invasively treated by sectioning the apical part of the implant using a transnasal approach.

## Consent

Since this is a case report, no approval of the Institutional Review Board was necessary.

Written informed consent was obtained from the patient for publication of this Case report and any accompanying images
